# Invisible medicine sellers and their use of antibiotics: a qualitative study in Cambodia

**DOI:** 10.1136/bmjgh-2019-001787

**Published:** 2019-09-20

**Authors:** Sovanthida Suy, Sonia Rego, Sothavireak Bory, Sophea Chhorn, Socheata Phou, Chanra Prien, Sotheara Heng, Shishi Wu, Helena Legido-Quigley, Johanna Hanefeld, Vonthanak Saphonn, Mishal S Khan

**Affiliations:** 1 Department of Public Health, University of Health Sciences, Phnom Penh, Cambodia; 2 Faculty of Public Health & Policy, London School of Hygiene & Tropical Medicine, London, UK; 3 Faculty of Pharmacy, University of Health Sciences, Phnom Penh, Cambodia; 4 University of Health Sciences, Phnom Penh, Cambodia; 5 Saw Swee Hock School of Public Health, National University of Singapore, Singapore

**Keywords:** health systems, public health, qualitative study

## Abstract

**Background:**

Global attention to antimicrobial resistance has increased interest in tackling the widespread inappropriate dispensing of antibiotics by informal, for-profit healthcare providers (HCPs). We provide new evidence on an understudied group of informal HCPs: invisible medicine sellers (IMS) who operate without any marked facility. We investigated factors that influence community decisions on which HCPs to purchase medicines from, focusing on reasons for using IMS, and compared different HCPs’ knowledge of antibiotic use.

**Methods:**

We conducted community focus group discussions (FGDs) in seven purposively selected villages representing high and low informal HCPs use in two peri-urban districts in Phnom Penh, Cambodia. Using information from the FGDs to identify HCPs that sell medicines, we interviewed 35 participants: 21 HCPs (including five IMS) and 14 key informants, including government HCPs and village leaders. We adopted an interpretative approach and conducted a thematic analysis.

**Results:**

Community members typically knew of several formal and informal HCPs selling medicines nearby, and IMS were common, as were doctors that sell medicines covertly. Two factors were most salient in influencing the choice of HCP for medicine purchasing. The first was trust in the effectiveness of medicines provided, judged by the speed of symptomatic relief. This pushed HCPs to provide several medicines, including antibiotics, at the first consultation. The second was the convenience offered by IMS and other informal HCPs: supplying medicines when other facilities are closed, accepting delayed payments, providing incomplete courses of medication and selling human antibiotics for animal use.

**Conclusion:**

This first study focusing on IMS indicates that it is important, but challenging, for public health agencies to engage with them to reduce inappropriate use of antibiotics. Although public health facilities must fill some gaps that informal HCPs are currently addressing, such as access to medicines at night, reducing demand for unnecessary antibiotics is also critical.

**Key questions**

**What is already known?**

- Private healthcare providers (HCPs), which include formal and informal providers, are widely used in low-income and middle-income countries, and are not well-regulated or monitored.
- Inappropriate use of antibiotics by informal providers is a global concern as this may be driving antibiotic resistance.

**What are the new findings?**

- Informal HCPs, including those that are ‘invisible’ because they have no marked healthcare facility, are commonly used for purchasing antibiotics in peri-urban Cambodia.
- They are trusted providers of medicines, despite communities knowing that they do not hold the required qualifications to sell medicines, because of their convenience and propensity to overmedicate to provide quick relief.

**What do the new findings imply?**

- Because communities want easy access to antibiotics, there remains a demand for services provided by invisible and informal HCPs that have inadequate knowledge of antibiotic use.
- It is challenging but important for public health agencies to engage with informal HCPs that are ‘invisible’.

## Introduction

Although there are opposing views on the optimal role of the private healthcare sector, it is clear that global constraints on government investment in public services are resulting in private healthcare providers (HCPs) playing a greater role in health service delivery to rich and poor populations.^1–4^ Private HCPs are used substantially more than public HCPs in many low-income and middle-income countries (LMIC). Their dominance is particularly pronounced in many Asian countries.^3–7^ This heterogeneous group encompasses traditional healers as well as allopathic ‘western medicine’ providers, of which some are highly qualified and officially licenced by the government (referred to as formal providers) while others do not have qualifications or licensing that is recognised by local authorities (referred to as informal providers).^3 8–10^

Evidence suggests that informal HCPs are the least trained and monitored group within LMIC health systems.^11^ Several studies from Asian LMIC indicate that informal HCPs lack the knowledge required to provide basic curative services, and a systematic review of 122 studies identified important recurring findings: informal providers typically show poor adherence to national guidelines, particularly in relation to inappropriate medicine provision, and their quality of care affects large populations since they are often a major component of the LMIC health systems.^10–14^ Despite the evidence on poor technical quality of care provided by informal HCPs, finding effective regulatory approaches in LMIC contexts continues to be incredibly challenging.^15 16^ Part of the reason for this is that regulatory interventions often do not adequately consider local health system dynamics and complexities, including two practical issues which we focus on in this study: first, some informal HCPs operate from locations that are difficult to identify, and second, there is often strong community demand for the services provided by informal HCPs.

### Invisible medicine sellers

**Figure 1:**
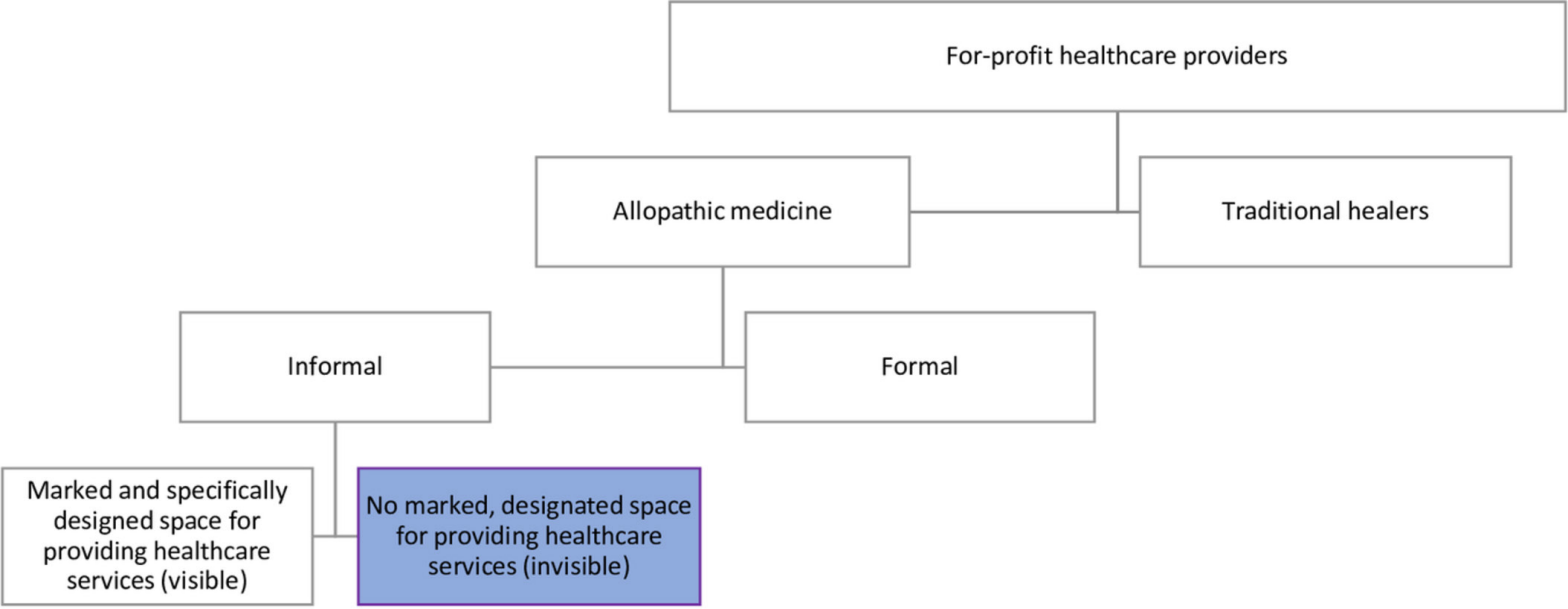
Position of invisible healthcare providers among different types of for-profit healthcare providers.

In this study, we further divide informal providers into two categories: visible HCPs, who provide health services within a physical space that is signposted and designed for health services, and invisible HCPs, who provide health services without having a marked and specifically designated outlet for this purpose (figure 1). Invisible HCPs have been insufficiently addressed by researchers and policymakers, largely because it is inherently difficult for authorities and researchers to identify them through standard surveys and inspections; therefore, their positive and negative contribution to local health service provision remains hidden. We specifically focus on invisible HCPs who engage in inappropriate dispensing of medicines, and who we refer to as invisible medicine sellers (IMS). Although any dispensing of medicines without establishing clinical need could pose a risk to patients, inappropriate provision of antibiotics is currently a major global concern owing to the scale of morbidity and mortality that is predicted to be caused by antimicrobial resistance (AMR).^17^ There is some evidence to suggest that for-profit HCPs, including IMS, excessively use antibiotics, and are driven partly by the desire to maximise profits and meet patient demands.^18–24^ However, no studies, to the best of our knowledge, have undertaken an in-depth analysis of IMS and their use of antibiotics in any country, including how IMS become trusted providers of medicine—thereby generating community demand for their services.

### Conceptualisations of trust in HCPs

As part of our analysis, we explored the concept of trust surrounding different types of HCPs, particularly those that are known to have no medical qualifications or licensing. Trust is a complex phenomenon rooted in subconscious thoughts that are difficult for individuals to recognise.^25^ Möllering conceptualises trust as a process consisting of three key elements: expectation, interpretation and suspension. He defines expectation as the outcome at the end of an interaction, which is preceded by the combination of interpretation and suspension. Interpretation refers to experiences that provide rational justification for trusting or not trusting a person or an institution. The third component, suspension, is the least explored; it refers to emotional bases of trust that move away from rational choices. This has also been referred by Simmel, who greatly influenced Möllering, as a mysterious sense of faith in another person.^26^ Throughout the paper, we differentiate between these three elements of trust, and will reflect on which of these are more present in the Cambodian context.

### Study setting and objectives

Cambodia is a lower-middle-income country with a population of approximately 16 million.^18^ Phnom Penh, Cambodia’s capital, is the most densely populated city in the country, with a population of 1.5 million. Phnom Penh is divided into 14 khans, composed of 953 villages. Each khan and each village has a leader, and clusters of villages are served by a local government-run health centre.

Cambodia’s public health system was weakened during the Khmer Rouge regime and the consequent Cambodian Civil War due to the systematic killing of intellectuals—including trained health professionals; consequently, the private health sector is very prominent.^27^ Nearly 70% of Cambodian patients first seek treatment in the private sector, and private medicine sellers are the preferred HCPs for the majority of those who are ill.^28^ The Medicines Policy of the Kingdom of Cambodia states that no medicines will be distributed through unauthorised outlets, and lays out clear regulations regarding sales of medicines: medicines are to be sold in pharmacies with trained pharmacists, and patients cannot obtain restricted medicines, such as antibiotics, without a prescription.^29^ Doctors are only allowed to sell a limited set of emergency medicines, although there is a lack of clarity around these rules.

Cambodia serves as a useful setting to study IMS and their potential role in AMR, as much of the action on unlicensed drug sellers in Cambodia has been triggered by concerns about AMR; this issue gained global attention when artemisinin resistance was discovered on the Cambodia–Thailand border in 2009, and resulted in high-profile ‘crack downs’ on unlicensed medicine shops until the number was reported to be close to zero in 2011.^30 31^ In 2014, Cambodia became one of the first countries in the Western Pacific Region to develop a national policy on AMR.^32 33^ However, drafting of the national action plan with concrete steps on addressing inappropriate use of antibiotics has been challenging since antibiotics are readily available without a prescription from formal and informal HCPs, and are sometimes substandard or false.^34–37^ Although information on licensed pharmacies is regularly updated by the Department of Drugs and Food in the Ministry of Health, the data only include those that are registered and there are reports of the continued existence and utilisation of unlicensed medicine shops.^38 39^ It is clear that licensed pharmacies are concentrated in the most central khans of Phnom Penh; unlicensed medicine shops are more prevalent in outskirts of the city, and remain the main source of care for those below the poverty line.^40–42^ Very little is known about the presence and role of IMS within this wider group of unregistered medicine sellers.

In light of the dearth of research on informal (particularly invisible) HCPs and their role as sellers of antibiotics, we investigated factors influencing the choice of HCP to purchase medicines for minor ailments, focusing particularly on reasons for using IMS, and on identifying different sources of trust in specific providers of medicine. We additionally compared HCPs’ knowledge of the correct use of antibiotics, seeking to understand whether IMS present a greater risk of inappropriately dispensing antibiotics.

## Methods

### Study population

**Figure 2:**
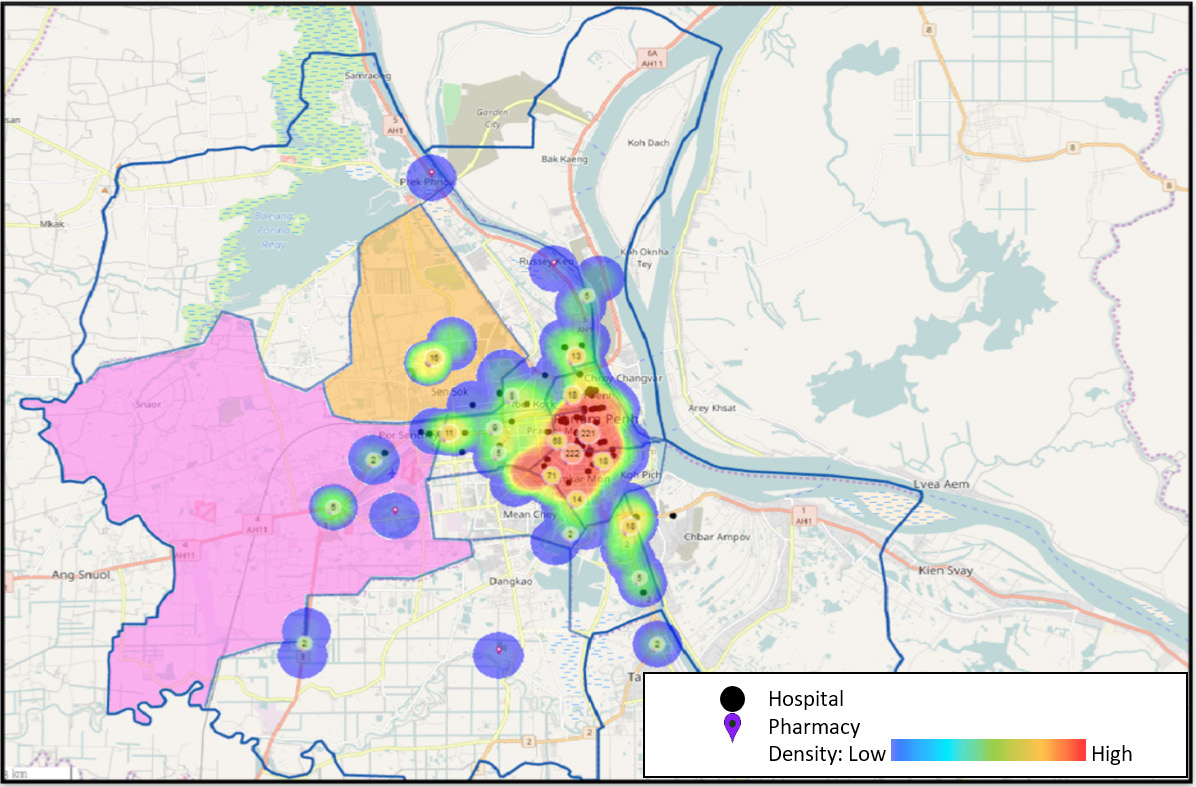
Map of hospitals and pharmacies in Phnom Penh with Khan Sen Sok (orange) and Khan Po Sen Chey (pink) highlighted.

This study was conducted in seven villages in two peri-urban khans on the outskirts of Phnom Penh: Po Sen Chey and Sen Sok. These khans, like others outside of the central part of Phnom Penh, have very few registered pharmacies, as illustrated in figure 2. Purposive selection of villages for the qualitative investigation was primarily based on data collected by a non-governmental service delivery organisation, Operation ASHA (OpASHA). OpASHA community health workers conduct door-to-door health screening, and as part of this screening, they undertook a rapid assessment of community healthcare-seeking behaviour. During the rapid assessment, five men and five women in each village were asked a series of questions including one about the use of informal HCPs for accessing medicines. We reviewed results of this rapid assessment across 122 villages in the study khans, and selected villages that represented low and high use of informal HCPs for buying medicines.^43^ Our selected villages represented a range such that 25%–80% of the respondents reported buying medicine from informal HCPs. Purposive selection also took the willingness of the village leader to support the study into account, in line with our ethical approval form. Village leaders contacted male and female participants and asked if they would like to participate in the focus group, which ensured a variety of occupations and age ranges.

### Data collection

Focus group discussions (FGDs) were conducted with community members from the seven selected villages. FGDs 1, 3, 4 and 7 were conducted in Po Sen Chey, and FGDs 2, 5 and 6 were conducted in Sen Sok. A topic guide was used to facilitate discussions on where to seek care when people are sick with minor ailments and participants’ views about types of HCPs. We worked as a group to translate and appropriately phrase questions in Khmer, and piloted the data collection process in one village. FGDs were led by an experienced bilingual facilitator in Khmer, who thoroughly explained who the researchers were and the purpose of their work, and two members from the research team (a mix of male and female researchers, with an MSc or PhD) observed the discussion and took detailed notes. No one other than participants and researchers was present during the FGDs. The research team worked with the village leaders to select 6–8 participants in each village, aiming for an even distribution of age and sex. FGDs were audio-recorded following consent, and typically lasted 45 min.

The details of every provider selling medicines that FGD participants mentioned were noted, including the location and demographic characteristics. Immediately following each FGD, the research team sought to locate these HCPs and approach them for an interview. We also interviewed public health centre staff, and khan and village leaders. The semi-structured interviews were conducted by two Cambodian researchers from our team of six (SS, SB, SC, CP, SH and SP), and were facilitated by a topic guide that explored perceived patient behaviour, referral processes, reasons interviewees think patients use different for-profit HCPs, knowledge of regulations related to medicine sales, and use of antibiotics.

### Qualitative data management and analysis

Interviews and FDGs were recorded and detailed field notes were taken, except when audio recording was not allowed by an interviewee. English language audio recordings were transcribed by a researcher (SR), and Khmer language audio recordings were translated and transcribed by external professional translators. After translation, Khmer language interviews were reviewed by University of Health Sciences researchers (SS and SC). Participants were de-identified and numbered in the transcripts. We conducted a thematic analysis employing an interpretive approach in which identified themes are supported by excerpts from the raw data to ensure that data interpretation remains directly linked to the words of the participants.^44^ This thematic analysis began by convening all authors closely involved in data collection (SS, SB, SR, MK, SP and SC) to discuss and agree on the emerging themes after reading the transcripts, then each transcript was coded line by line in NVivo (V.12). A constant comparison method was adopted to identify what characteristics participants valued when choosing HCPs for accessing medicines. By comparing what they reported valuing, we were able to identify characteristics they specifically appreciated in IMS and what characteristics they valued in other providers but were missing in IMS. Thematic saturation was established when the research team discussed and agreed that no new themes were emerging from the data. Each excerpt indicates whether it is taken from an FGD or interview with visible medicine seller (VMS), invisible medicine seller (IMS) or key informant (KI).

### Ethics

Written informed consent was taken before conducting interviews or FGDs. Since some of the HCPs we interviewed may have been contravening regulations by selling medicines, we do not name the villages selected for data collection to protect the anonymity of respondents.

### Patient and public involvement statement

Patients/the public were not involved in designing this study. We plan to produce a dissemination presentation to help to disseminate the findings to the public.

## Results

The first step in our study, analysis of the rapid assessment of community healthcare-seeking behaviour with respect to informal providers, indicated high use of IMS; we found that use of IMS was reported in 62% of all villages in the study khans. A total of 40 women and 20 men aged between 27 and 85 years participated in the 7 community FGDs. In line with the rapid village survey, most FGD participants knew of IMS in their vicinity, although some participants in FGD 2 and 7 said that medicine sales by IMS no longer occurred in urban or peri-urban areas—including in their communities—and they believed that this practice is now limited to rural areas. In contrast, in FGD_4, an elderly woman openly spoke about herself and a relative selling medicine from their homes and in FGD_6, a female said that her grandmother sells medicines from home. We observed differences in opinion about the availability of medicines, such as antibiotics, from IMS within the same FGD.

Maybe at rural area (there are) grocery houses which sell some medicine. But we do not have that type of places here. (FGD_2)

They secretly do it. Sometimes people carry and sell around at the market and we do not know which they are and what sources they are from. (FGD_2)

We located ten IMS in the seven study villages (in addition to the woman in FGD_4 who sold medicines herself), of which five agreed to be interviewed. Only one of these IMS claimed to have relevant training (nursing); all of the others had loose links to the medical profession but did not have qualifications that would equip them to sell medicines. We identified a further 16 visible HCPs who sold medicines in the same villages, and interviewed all of them. Information about interviewees is summarised in table 1.

**Table 1:** Summary of interviewees in Khan Sen Sok and Khan Po Sen Chey

VMS: Visible medicine sellers (n=16; 10 females and 6 males)	Five pharmacists; one doctor running a pharmacy; two doctors who were selling medicines from their clinic; five medicine sellers, of which two were running outlets owned by doctors; one midwife; one nurse; one unqualified seller whose sister is a pharmacist (all qualifications were self-reported and were not verified)
IMS: Invisible medicine sellers (n=5, 4 females and 1 male)	One guard at a health centre; one seller who stayed with doctors during civil war; one former pharmaceutical sales representative; one seller whose sister took a pharmacy short course; one nurse
KI: Key informants from study villages (n=14; 5 females and 9 males)	Four government health centre staff; four OpASHA community health workers; six village leaders/deputies

OpASHA, Operation ASHA.

Although our study focused on IMS, we found that doctors—who are only allowed to stock medicines required for emergency care according to Cambodian regulations—often (secretly) sold a range of medicines, including antibiotics from their clinics, thereby also acting as medicine sellers that are difficult to identify. We, therefore, first present findings about factors influencing the choice of HCP to purchase medicines for minor ailments, focusing particularly on reasons for using IMS, and on different sources of trust in specific providers of medicine. In the final section, we report on HCPs’ knowledge of the use of antibiotics, and their views on regulations around medicine sales.

### Factors influencing the choice of HCP to purchase medicines from

#### Trust in the effectiveness of the medicines provided

The strongest theme emerging in all FGDs and interview with some community health workers (KI_1, 2 and 3) was that when a person believed that medicines from a specific HCP were effective, they were likely to revisit the provider, regardless of cost, qualifications of the provider or distance, and were likely to recommend the provider to others. Furthermore, several participants in FGD_2 and 3 noted that if one HCP’s medicines did not cure them, they would go to a different one without delay.

For me, I went and got the medicine and I got cured. For example, when I got swollen leg or headache, I brought medicine there and it was effective. So it becomes belief. And if it is not effective, I find another one. (female participant in FGD_2)

#### Quick access to medicines at times when other health facilities are closed

Access to medicines at times when other health facilities are closed was a recurring concern among FGD participants (FGD_2, 3, 5 and 6); this was dealt with by either approaching IMS when there was a need for medicine at night or by keeping a supply of medicines at home for themselves and their friends or family. A female FGD participant, who voluntarily offered information about selling medicine from home, explained that she plays an important role because people come to her when they get sick at night (FGD_4). It was also reported that community members like to visit HCPs who sell from their homes because, unlike public health facilities, the HCP is present at all times and they do not have to wait (FGD_3).

#### Being able to get medicines even with limited means of payment

Some accounts from FGD participants and HCPs indicated that people struggle with being able to purchase the full course medicines. In FGD_4, some participants spoke about using IMS because they will allow them to take medicines with partial payment and owe the IMS money until they can pay. In keeping with this theme, it was clear that several HCPs—visible and invisible—perceived a demand from patients for medicines to be sold in small quantities due to the lack of means to pay, and that more qualified providers, such as pharmacists, may be reluctant to sell incomplete doses (VMS_9, IMS_11 and 15).

For those who have money, they buy drugs for two doses and the poorer one only buys drugs for one dose. (IMS_11)

Even if there is prescription asking them to take drugs from 7 or 10 days, some would ask to buy drugs for like only 2 days … It does not matter how (much) I explained the situation to them … But when we don’t sell drugs to them, they will go and buy drugs from other places. (VMS_9, a qualified pharmacist running his own pharmacy)

#### Leniency in selling medicines to be used for animals

Another theme that emerged from a subset of the interviews was the use of HCPs that provide medicines for animals, as well as humans. Several sellers in medicine shops (IMS_2, VMS_3, 4, 7, 19, 20 and 21) and some community health workers (KI_1) said that people come in to buy medicines for their cattle, chickens and dogs. Two of the sellers (VMS_3 and 20) went on to elaborate that most medicines sold for animals were for chickens involved in cockfighting, and another reported that the majority of his clients purchase antibiotics over-the-counter for their cattle and chickens.

#### Other factors influencing the choice of provider for purchasing medicines

Other factors influencing the choice of provider for purchasing medicines for minor ailments that we probed about were: cost of medicines or consultations, distance to travel to access HCP, level of medical qualification and size of health facility. These factors emerged as less salient in influencing decisions, and trust in the effectiveness of the medicine provided was the dominant consideration, even when cost and qualification of the HCP were specifically probed about.

I do not think whether the places belong to the public, the private or license or non-license. Only (whether) it is effective or not, then I will go and buy. I do not care about the price, either cheap or expensive, registered or not registered. (FGD_4)

### Reasons for trust in the effectiveness of medicines provided by the chosen HCP

We further analysed different reasons for developing trust in the medicines supplied by an HCP, as this was a very common consideration that FGD participants referred to.

#### Patient or family members’ experience during a recent illness

Trust in the effectiveness of medicines appeared to be most closely linked to how quickly the individual or a family member experienced symptomatic relief during a recent illness, with FGD participants (FGD_3, 5 and 7) repeatedly using phrases such as ‘cured faster’ and ‘reduce pain in short time’ to describe effective medicines (usually referring to antibiotics). Consistent with this finding, we noted that some HCPs (VMS_9 and 7) were acutely aware of the strong patient demand for rapid relief and propensity to shop around. For example, one trained pharmacist (VMS_9) explained that he felt forced to dispense antibiotics because patients would accuse him of trying to prolong the illness to make more money if he gave other medicines that were perceived to be less effective, and felt that patients trust the opinions of friends and family more than the advice of the HCPs.

When the medical experts tell them to do the right thing, the patients don’t do and don’t believe in it. The people trust their peers more…. (VMS_9)

#### Level of training or qualification

There were contrasting findings on the extent to which trust in a HCP was related to whether he or she held a formal qualification. Participants in several FGDs indicated that people generally prefer to buy medicines from qualified providers (FGD_1, 3, 4 and 5), usually because of concerns around of receiving the wrong medicine from HCPs who had not been adequately trained. The specialist training received by doctors and pharmacists meant that they were perceived to hold greater skills in correctly diagnose illnesses (FGD_2, VMS_3 and 14, KI_6 and 9). Doctors were valued because they can ‘check the patient’s health’ before prescribing (FGD_5, KI_12) and know which medicine is correct for the symptoms (KI_9). One participant explained that medicine ‘is like a razor’, referring to the belief that it can have positive or negative effects depending on how it is used (FGD_1). It appeared that community members may look for a ‘proper sign’ or other visual cues to indicate that the seller is appropriately trained (FGD_5 and 6).

They should have sign or logo approved by the Ministry of Health. They should wear white dresses to sell medicine. (FGD_6)

However, others said that they had trust in medicines provided by those who had learnt from experience just as much, or more, than an HCP with formal qualifications (FGD_3, 4 and 7).

Drug sellers are different. One has skill(s) from the school. Another one gets skills from experience. They can sell medicine from their learning and sharing with others. (FGD_3)

#### Access of HCP to informal sources of knowledge

Our analysis indicated that there were two main factors that allowed IMS to gain legitimacy and trust within communities as HCPs that sell effective medicines without being based in a health facility. The first was access to knowledge about medicines from family members or friends with relevant qualifications, such as a sister who has completed some pharmacy training (IMS_2). A participant in FGD_3 illustrated how an invisible medicine seller cooperates with a doctor and how this leads to her being perceived as a provider of effective medicines.

[the invisible medicine seller] told the physician to write prescriptions and put each in each pack for different disease treatment. At night when someone knocks on her door and asked for diarrhoea, then she hands over a pack of medicine for diarrhoea. Before handing over the medicine to the patient, she looks at the prescription in the bag to make sure that the words says for diarrhoea. Such drug seller is smarter than a trained seller. (FGD_3)

The second factor identified was informal medical training or experience, including experience during the Cambodian Civil War (IMS_11, FGD_4); learning through of their primary job working as ancillary staff in a hospital or health department (IMS_10, KI_10); or knowledge provided by pharmaceutical companies (KI_7, IMS_12).

#### Having a personal connection with the HCP and knowing that an HCP works in a government hospital

Finally, some participants in FGD_3 and 5 indicated that knowing an HCP personally can help to develop a trust in the medicines they provide, and several FGD participants and a community health worker indicated that HCP working in a government facility helped to attract patients to a side business, selling medicines in the evening (FGD_1, 3, 4, 5 and 6; KI_1).

##### HCPs knowledge about antibiotics

Our study revealed potentially harmful views about the use of antibiotics among visible and invisible medicine sellers alike, including the wide range of illness antibiotics should be used for, and how to determine the dosing to use. Beliefs about the frequent need to use antibiotics were often related to the conceptualisation of antibiotics as medicines that heal a range of ailments (the Khmer word for antibiotics, Thnam Phsas, means ‘heal wound’) and that antibiotics are safe relative to other medicines (VMS_6, 10 and 14, IMS_11).

The greatest detail about the (mis)use of antibiotics was provided, with confidence, by a female invisible medicine seller who learnt about medicines during the Khmer Rouge regime (IMS_11).

… ampicillin, amoxicillin, penicillin, used to put on wounds … we break them into small pieces and pour them on wounds on our legs. I have been in the business for quite a long time. That’s why I know. (IMS_11)

If you are very sick, you can take it (ampicillin) three times per day. If you are not that sick, you can take it for two times. I tell them that they must buy mixed drugs or the drug will only cure one symptom. You won’t cure diseases. For diseases like coughing, if you only take drugs for coughing without antibiotics, you won’t get better. (IMS_11)

This seller was aware of the need to refer certain patients to the hospital, but felt that antibiotics were safe to her to sell. Similarly, the female invisible medicine seller identified during FDG_4 appeared to have limits around the number of medicines she felt confident selling, and antibiotics (ampicillin) was one of them.

Other IMS we interviewed were more reserved, but did display misconceptions, such as the need to always use antibiotics when someone has a cough (IMS_2) or diarrhoea (IMS_10), and openly admitted that they provide medicines based on patient demand (IMS_10, 12 and 15). The propensity to use antibiotics at the first sign of common illnesses, such as cough or ‘inflammation’, was also found among unqualified VMS (VMS_6, 19 and 20), as was selling of antibiotics in response to patient demand (VMS_3 and 5).

Mostly (best sellers are), Amok and Ampi. They mostly buy Amok and Ampi to use because they know these drugs. (VMS_3)

Some of the HCPs did share ideas about appropriate use of antibiotics and knowledge about antibiotic resistance, referring to only selling a full dose (VMS_3), informing patients of the importance of completing their full course of treatment (VMS_4 and 6), mentioning the link between incomplete treatment and drug resistance (VMS_4, 5, 6 and 7) and explaining that antibiotics should not be used of minor illnesses, such as a cold (IMS_12, VMS_9), or to obtain profits (VMS_5 and 9).

In general, we found that doctors and trained pharmacists tended to focus on their responsibilities to patients and to avoid overprescribing, whereas IMS spoke in terms of providing medicines according to the patients’ request.

### HCPs knowledge of and views on regulations around medicine selling

Most HCPs we interviewed, with some exceptions (IMS_11, VMS_17), were aware of regulations on who is allowed to sell medicines, such as antibiotics. We found two opposing views about whether existing rules that restricted sales of controlled medicines, such as antibiotics, to qualified HCPs were appropriate. Some sellers (VMS_6, 7, 9 and 14, IMS_11) acknowledged that they were not allowed to sell medicines, but justified their practices based on pragmatic considerations, and argued that the rules should allow for a wider set of providers (those who have done short courses or have relevant experience) to sell medicines legally. For example, a doctor who was open about selling medicines against regulations explained that after diagnosing a patient ‘(if) I don’t have drugs to sell to them, it does not make sense’ (VMS_7).

The other dominant view was that pharmacy training should be a minimal requirement to run a medicine shop (VMS_3 and 9, IMS_10), and that other types of training or on-job experience was not sufficient.

They do a short course, they are drug sellers. Yes, they can sell but it is not fair. Why? Because we spend 5 years or more and they spend only a few months and they also can sell. Their knowledge is very different. (VMS_3)

## Discussion

We provide new information about challenges faced by regulatory authorities in LMIC tasked with implementing rules that restrict access to antibiotics through licensed providers, specifically challenges related to the presence of HCPs that are difficult for people outside of the community to locate. Our study in peri-urban Cambodia revealed that IMS are common, they sell antibiotics often with dangerous misconceptions, and are trusted by communities as providers of effective medicine. These findings are relevant beyond Cambodia, as challenges to implementing restrictions on access to antibiotics through informal providers are salient in many LMIC.^45 46^

In all of our study villages, there were multiple HCPs that residents could buy medicines from, and we identified two broad factors—trust in the effectiveness of medicines provided and convenience of accessing the desired medicines—that were most salient in influencing the choice. Trust emerged as a salient theme in our analysis. Most participants reported trusting HCPs based on how quickly the medicines provided were perceived to alleviate symptoms, and qualifications or formal licencing were usually secondary considerations. We found that this put pressure on HCPs to give multiple medications, including antibiotics, at the first consultation. Overmedication by providing a combination of medicines that cover a range of symptoms—also known as polypharmacy—has also been found in other studies conducted in Cambodia^36 47–49^; our findings further indicate that informal HCPs’ access to, and use of, multiple medications at the first consultation may allow them to address symptoms of common illnesses without having any medical knowledge, thereby building trust in them and gaining popularity.

Relating our findings to Möllering’s conceptualisation of trust, we identified rational justifications based on perceptions of effective symptomatic relief, which link to his two elements of expectation and interpretation. We also identified his third component known as suspension, as some community members indicated an emotional basis of trust associated with having a personal connection to the HCP.

Apart from trust, the main reason for using IMS is the gap they fill in existing health services by supplying medicines at night time, being flexible in taking delayed payments, giving incomplete courses of medicines when patients cannot afford a full course and knowingly selling human antibiotics for animal use. These are the features demanded by patients but rarely available in formal or visible HCPs.

Although we identified a substantial number of IMS in our study villages, and successfully implemented a novel methodology for doing so, we acknowledge that the number of interviews we were able to conduct with IMS was small; this was partly because half of the IMS we approached were not willing to be interviewed, which was unsurprising, due to the hidden nature of their work. We believe that a strength of our study was the use of highly skilled Cambodian researchers who were able to work with community members to find local IMS, and were able to get consent from five IMS for an interview. Another limitation of this study is the potential desirability effect: community members and HCPs may have answered in a way that they believed made their views more favourable to researchers or other participants. It is likely that IMS are found more frequently in rural areas,^50^ and our results may underestimate the presence of and reliance on IMS. Future studies in rural Cambodia may provide an insightful comparison with our results.

## Conclusion

Although IMS are difficult for public health agencies to locate for engagement activities, it is important to address inappropriate dispensing of antibiotics by this group of HCPs. To reduce community use of informal HCPs that facilitate inappropriate access to antibiotics, public health services must fill some of the gaps that these providers are currently addressing, such as access to medicines at night. However, in taking steps to improve appropriate access to antibiotics, public health services will not and should not provide antibiotics primarily to meet patients’ expectations. Therefore it is also critical to reduce community demand for unnecessary antibiotics.
